# Breast Cancer in Developing Countries: Opportunities for Improved Survival

**DOI:** 10.1155/2010/595167

**Published:** 2010-12-29

**Authors:** Lawrence N. Shulman, Walter Willett, Amy Sievers, Felicia M. Knaul

**Affiliations:** ^1^Dana-Farber Cancer Institute, 44 Binney Street, Boston, MA 02115, USA; ^2^Department of Nutrition, Harvard School of Public Health, 665 Huntington Avenue, Boston, MA 02115-6018, USA; ^3^Harvard Global Equity Initiative, 651 Huntington Avenue, Boston, MA 02115, USA

## Abstract

Breast cancer survival in the USA has continually improved over the last six decades and has largely been accredited to the use of mammography, advanced surgical procedures, and adjuvant therapies. Data indicate, however, that there were substantial improvements in survival in the USA even prior to these technological and diagnostic advances, suggesting important opportunities for early detection and treatment in low- and middle-income countries where these options are often unavailable and/or unaffordable. Thus, while continuing to strive for increased access to more advanced technology, improving survival in these settings should be more immediately achievable through increased awareness of breast cancer and of the potential for successful treatment, a high-quality primary care system without economic or cultural barriers to access, and a well-functioning referral system for basic surgical and hormonal treatment.

## 1. Introduction

Breast cancer is a leading cause of death and disability among women, especially young women, in low- and middle-income countries [1]. Though incidence and overall mortality rates continue to be lower than in most high-income countries, case fatality rates from breast cancer are very high. These high case fatality rates are likely due to a lack of awareness of the benefits of detection and treatment and a scarcity of adequate facilities for detection and diagnosis, as well as poor access to primary treatment. 

Remarkable improvements have been achieved in the probability of survival for women diagnosed with breast cancer in the USA as compared to 60 years ago [2]. Early detection through the use of mammography, high-quality surgery, and adjuvant therapies including chemotherapy and targeted therapies, such as hormonal therapy and, more recently the HER2-directed agent trastuzumab, can be credited for much of the recent improvement in outcome for women with breast cancer in the USA. However, even prior to the routine use of mammography or adjuvant therapy, significant improvements were made in breast cancer survival, and these can be traced to relatively low-cost interventions that are still in use in high-income countries. Understanding which healthcare interventions were available and how they resulted in improvements in the probability of survival could be important, especially for designing programs in resource-constrained settings where breast cancer case fatality is high and many of the most costly and technology-intensive diagnostic and therapeutic options are not available. 

## 2. Breast Cancer in Low- and Middle-Income Countries

In many developing countries, the incidence of breast cancer is now rising sharply due to changes in reproductive factors, lifestyle, and increased life expectancy. Today, more than half of incident cases occur in the developing world [14, 15]. Combined with still high case-fatality rates, this means that mortality from breast cancer is a leading cause of death among adult women in developing countries, as well as in the developed world. In Mexico, for example, breast cancer is now the second leading cause of death among women aged 30 to 54 and the leading cause of tumor-related death among adult women of all ages [16]. 

The high probability of dying from breast cancer—the case fatality rate, which is approximated by the ratio of mortality to income—across the developing world further reflects the inequities in early detection and access to treatment [1, 17]. The number of deaths as a percentage of incident cases in 2008 was 48% in low-income, 40% in low-middle-income, and 38% in high-middle-income countries, while it was 24% in high-income countries according to the most recent Globocan/IARC data [18]. 

Available evidence on stage at diagnosis, though scarce, indicate that a very high proportion of cases in the developing world are detected in late stages [1, 3]. (Table 1) In many underserved populations, a majority of women present with advanced disease; the figure is as high as 78% in black women in South Africa. In contrast, in the United States the majority of cases are detected in localized stages of the disease (Stages I and II), a third is regionally advanced (Stage III), and only 5% are distant-stage metastatic (Stage IV) [13].

Many reasons are given for the advanced stage at presentation and resultant poor survival rates in low- and middle-income countries: the stigma of breast cancer and the associated societal implications of its treatments (especially mastectomy) discourage women from seeking care early on; lack of knowledge about breast health; scant options for early detection due to limited access to routine care and examinations; and lack of access to mammography and to affordable, high-quality treatment options. 

## 3. Opportunities to Improve Breast Cancer Outcomes for Women in Developing Countries

In the short term, mammography and other expensive and technologically complicated resources and therapies will not likely be available to many of the world's women. Though we must continue to work at all levels to bring diagnostics and therapeutics with a proven impact on outcomes to these women as soon as possible, there are ways closer at hand to improve the immediate outlook for women in these settings. 

**Figure 1:**
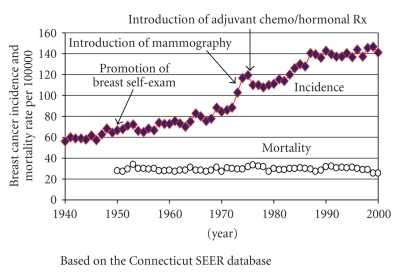
Breast cancer incidence and mortality, USA, 1940–2000.

Figure 1 shows the incidence and mortality rates for breast cancer in the USA between 1940 and 2000. From the late 1940s, breast cancer incidence rose steadily. By contrast, mortality rates did not rise appreciably during this period. Thus mortality-to-incidence ratios decreased dramatically, even before the generalized use of mammography or adjuvant chemotherapy and antiestrogen therapy that commenced in the mid- to late 1970s. 

Table 2 presents the ratio of mortality over incidence, as an approximation of the case-fatality rate, in 5-year increments between 1950 and 1975. Between 1950 and 1975 incidence nearly doubled, increasing from 66.6/100,000 women to 119.2/100,000, while mortality remained relatively constant, 28/100,000 and 31.6/100,000, respectively. Thus, during this time period, the ratio of mortality over incidence (an approximation of the case-fatality rate) fell from 0.42 to 0.27 representing a 36% decline: This suggests that more women were surviving their cancers in 1975 as compared to 1950 and is true for both whites and blacks [19]. Further, the reduction in case-fatality rates is at least as large as the improvement evidenced since the introduction of mammography and adjuvant therapy. These findings suggest considerable room for reducing the high mortality-to-incidence ratio found in many developing countries even without mammography or adjuvant therapy. 

## 4. Explaining Improved Breast Cancer Survival Rates in the USA Prior to 1975

The increases in incidence and survival for breast cancer in the USA between 1950 and 1975 cannot be attributed only to detection of *in situ* cancers that would not have progressed. The proportion of *in situ* cases in known-stage cases in the Connecticut Tumor Registry in that period was very small and increased from only 0.3% in 1950–1954 to 1.9% in 1970–1974, and it was largely unaffected by improved reporting and a reduction in unknown-stage tumors. Thus, the reduction in the mortality-to-incidence ratio must largely reflect outcomes for patients with invasive cancers. From 1940 to 1970, the stage distribution of reported cases in Connecticut improved substantially. Regional and advanced stages fell from 58% to 54% between 1940–1944 and 1950–1954, and to 45% in 1970–1974 [20].

The period from 1940 to 1974 was a time in the USA when evidence-based medicine became more widespread, and healthcare became more generally available, including increased use of routine gynecologic and general physical examination. Cancer and the human breast also became acceptable topics of conversation. For example, the American Cancer Society began promoting self-examination for breast cancer in 1950 [21] and routine screening by cervical cytology starting in 1952 [22]. Further, the era of oral contraceptives in the 1960s contributed to greater interactions between healthy women and their healthcare providers. Authors who analyzed data prior to 1974 assign the improvements in survival to more effective breast education programs, increased breast cancer awareness, detection of tumors palpable with self or breast-clinical examination, and better diagnostics [19, 20]. Thus, the increase in survival rates in the USA prior to 1975 strongly suggests potential to improve breast cancer outcomes in developing countries more quickly than we will be able to make routine mammography and adjuvant therapy available. 

Recent studies, showing breast physical examination and breast self-examination to be unhelpful in reducing stage at diagnosis [23–26], have considered only developed countries or urbanized areas of developing countries where routine healthcare is generally available, breast cancer awareness and education are high, and mammography is more routinely accessible. These data, and hence the findings, are likely to be less applicable to a population where breast cancer education and awareness are low, access to the healthcare system severely restricted, and the vast majority of patients present with advanced disease. 

## 5. Next Steps to Improve Breast Cancer Survival in Low- and Middle-Income Countries

While reducing the incidence of breast cancer is an ideal goal, the options for achieving this are limited and longer term, particularly for the developing world. Healthy lifestyle, including limiting alcohol consumption, maintenance of ideal body weight, regular physical activity, and avoidance of postmenopausal hormone replacement therapy, can have an important impact on breast cancer incidence [15, 27]. Every effort should be made to limit these risk factors and thus breast cancer risk. Yet, even with strong efforts aimed at prevention, the incidence of breast cancer is likely to increase in most developing countries due to changes in reproductive patterns including later first pregnancies, reductions in parity, and shorter duration of lactation; as well as, declines in physical activity and increased life expectancy. 

Increasing survival rates should also be a priority. Earlier detection and timely, adequate surgery would likely result in substantial improvements in survival in much of the developing world. Education about breast cancer, advocacy around curability, and increased coverage of basic healthcare including skilled breast physical examinations could produce improvement in survival rates as occurred in the USA between 1950 and 1975.

Education efforts need to address the reality that many women, particularly those with less income and education, may not seek care when they feel a breast mass, because they are unaware of what it represents, are concerned about the stigma of cancer and being rejected by their community and their partners, fear the potential loss of the breast, or believe there are no effective therapies for the disease especially if all the women they have known with breast cancer died. HIV—a stigma-laden disease, that if untreated is universally fatal—provides important lessons [28]. These same issues prevented many patients with HIV from seeking care. By contrast, it has been demonstrated that by combining education, with better and more accessible healthcare facilities, trained medical personnel, and effective therapy, patients do seek and comply with treatment and benefit from it [28–30].

The ability to provide adequate affordable access to physical exams by healthcare workers is not a trivial obstacle. An essential first element is the existence of a functioning primary care system staffed by providers trusted by their community. While many countries continue to battle with a weak primary infrastructure, examples, such as the *Oportunidades* program and *Seguro Popular* in Mexico and Partners In Health in rural Africa and Haiti, provide important lessons for strengthening primary healthcare including, and often especially, interventions to improve the health of women [16, 31, 32]. 

These interventions are essential parts of overall health system strengthening and can help with the prevention and treatment of many diseases in addition to breast cancer. Clinical breast exams do not need to be performed by physicians or nurses. In settings where community healthcare workers have learned to care for patients with diseases as complex as HIV, multidrug resistant tuberculosis, and malaria, they could be trained to effectively perform breast exams. 

Large-bore core needle biopsy is a reliable method to obtain tissue for diagnosis and can be performed by trained personal in relatively simple ambulatory settings. Ultrasonography, widely available in developing countries, can effectively localize tumors for biopsy. Pathology services must be available to process the specimens but can be located regionally or outsourced globally. 

In many developing countries, surgery is available in regional centers, although additional training of surgeons in appropriate techniques may be needed, and women will require financial support and transportation. Where radiation therapy is not available, as is the case in many low-income countries, the surgery should be a mastectomy.

Given the high proportion of hormone receptor positive cancers, tamoxifen can be effectively combined with surgery. Unlike many treatments for breast cancer, generic tamoxifen is low cost, taken orally, and in the vast majority of patients is well tolerated and does not generate unmanageable side effects or require additional medications or care to control symptoms.

## 6. Conclusions

Options exist to greatly expand low-cost alternatives for earlier detection and treatment of breast cancer in developing countries. Guidelines have been developed and have been stratified according to the resources available in specific countries and health systems [33–36]. Many of the basic interventions focus on education, awareness building, the health of women, and expanding capacity at the primary and community healthcare levels, and thus and also contribute to overall health system strengthening [37]. 

Education to improve breast health awareness, breast self-examination, and clinical breast exam are relatively inexpensive and can be incorporated into existing primary health infrastructures. Surgery and hormone therapy based on tamoxifen are cost effective, especially with early detection, and implementable in poor-resource settings. Focusing on providing these interventions in locations where they do not currently exist could dramatically improve survival. 

In no way does this abrogate the responsibility to eventually provide resources such as mammography, adjuvant chemotherapy, and advanced targeted therapies such as trastuzumab in these settings. However, great benefit can emerge from basic breast cancer education and awareness, integrating breast exams into primary healthcare infrastructure, and adequate surgery combined with tamoxifen. Implementation of these interventions should proceed as quickly as possible, while the more complex and costly interventions, such as mammography, are being made more available. The provision of better primary healthcare, education, and better medical outcomes will provide a solid foundation for reducing stigma and fear that will make more effective the introduction of complex technologies, such as mammography or adjuvant therapy. 

There is no reason not to immediately strive for the implementation of basic interventions for breast cancer care and control in all settings. It is, in fact, our obligation. 

##  Competing Interest Statement

All authors have completed the Unified Competing Interest form at http://www.icmje.org/coi_disclosure.pdf (available on request from the corresponding author) and declare that all authors had: (1) no financial support for the submitted work from anyone other than their employer, (2) no financial relationships with commercial entities that might have an interest in the submitted work, (3) no spouses, partners, or children with relationships with commercial entities that might have an interest in the submitted work, (4) no nonfinancial interests that may be relevant to the submitted work.

## Figures and Tables

**Table 1:** Stage of initial diagnosis of breast cancer for a selection of low- and middle-income countries and USA [3].

Region	Country and city	% Stage I/localized	% Stage III-IV/regional-metastatic	Year (s)	Source of data
Latin America	Mexico [4]	14	48	2002	Registry of the Mexican Social Security Institute
	Peru, Lima [5]	9	49	1985–1997	Instituto Nacional de Enfermedades Neoplasicas
	Brazil, [5]				
	Sao Paulo	10	67	1979–1989	Academic Hospital of the University of Sao Paulo
	Puerto Alegre	16	30	1975–1997	Academic Hospital of the Fed. University of Rio Grande do Sul

Asia	India: [6, 7]				
	Mumbai	8	35	1995	Tata Memorial Hospital Registry
	Trivandrum	4	53	1996	Hospital Cancer Registry Trivandrum

Middle East	Saudi Arabia [8]*	24	62	2004	National Cancer Registry
	Jordan, Amman [9]**	23	37	2008	Jordan Cancer Registry
	Egypt, Gharbia [7, 10]*	26	74	2000–2002	Tanta Cancer Registry
	Egypt, South [11]	11	50	2001–2008	South Egypt Cancer Institute

Africa	South Africa [12]				
	Blacks	5	78	1970–1987;	Grote Schuur, Cape town;
	Whites	31	31	1976–1997***	Provincial Hospitals of Port Elizabeth and East London, and Johannesburg General Hospital

North America	United States [13]	60	38	1999–2005	National cancer registry

*For these countries data were not provided by stage (I, II, III, IV) and were given only as localized versus regional or distant metastatic.

**The Jordan Cancer Registry for 2008 figures are 3% in-situ, 23% Stage I, 29% Stage II, 23% Stage III, 14% Stage IV, and 7.5% unknown.

***Data collected from 4 hospitals, three from the first-time period listed and the fourth from the second-time period listed.

**Table 2:** Mortality/incidence ratios for breast cancer in the USA between 1950 and 1975.

Year	Incidence/100,000	Mortality/100,000	Mortality/incidence ratio
1950	66.6	28	0.42
1955	64.9	29.8	0.46
1960	73	28.3	0.39
1965	82.9	28.5	0.34
1970	84.4	27.4	0.32
1975	119.2	31.6	0.27

Based on the Connecticut SEER database.
